# Pareto-critical equilibrium condition for non-linear multiobjective optimization problems with applications

**DOI:** 10.1016/j.heliyon.2024.e39498

**Published:** 2024-10-22

**Authors:** Shakoor Muhammad, Abdul Rehman, Amjad Iqbal, Taimur Ali, Faisal Khan

**Affiliations:** a Department of Mathematics, Abdul Wali Khan University, Mardan 23200, Pakistan; b Department of Mathematics, University of Balochistan, Quetta 87300, Pakistan; c College of Engineering and Informatics, National University Ireland, Galway 91 CF50, Ireland

**Keywords:** Multiobjective optimization, Pareto-critical equilibrium condition, Downlink transmit beamforming, Multi-agent system

## Abstract

The new (necessary) Pareto-critical equilibrium condition (PCEC) discussed in this study is based on the Pareto-critical condition for non-linear optimization problems. The proposed condition is evaluated by defining a multiobjective optimization problems (MOOPs) subject to a set of constraints. Then this MOOPs is converted to a single objective optimization problem by using the available optimization techniques, for instance, the weighted sum method, and solved by the non-linear Nelder-Mead simplex method. The obtained solution points satisfy the proposed PCEC of an unconstrained multiobjective optimization problem (MOOPs) under the convexity assumption. This PCEC is evaluated in a search that either results in a point inside the feasible region or a point for which the PCEC holds. The resulting Pareto-critical equilibrium condition is globally valid for convex problems. Numerical experiments are carried out in order to clarify the proposed condition's validity. Moreover, the proposed PCEC is used to place a downlink transmit beamforming system base station in an ideal position as a first application and is also employed in situations where there are multiple agents working towards a common goal as a second application.

## 1 Introduction

In many practical applications, the usage of the equilibrium point attempts to provide a solution to the question of what will likely happen if many people are sharing limited resources and everyone is selfish. This circumstance is particularly significant in problems where multiple persons are involved in the task. Multiobjective optimization problems (MOOPs) seek the best compromise among conflicting objectives. Pareto optimal solutions offer the best trade-off, forming the “champion set” known as the Pareto optimal set (P). These solutions satisfy the “Pareto-criticality conditions,” ensuring no objective can be improved further without sacrificing another. While the Pareto critical set encompasses all candidates meeting these rules, the optimal set is the elite group of true champions who excel in all aspects, achieving the most balanced outcome. According to [1], it was the infeasibility of a non-linear optimization problem as a Pareto-criticality condition of an auxiliary multiobjective optimization problem. For non-linear programming problems an infeasibility certificate is established [1] based on the pareto critical condition. When it comes to convex nonlinear optimization problems this condition becomes sufficient. Multi-objective optimization problems can be successfully solved by using the Pareto critical set [2]. For the self-diffusion of silicon in thermally produced silicon dioxide, the equilibrium condition is utilised [3]. For the distance of convex functions based on the Pareto critical condition, a proximal point approach has been established [4]. When a problem is infeasible, the primal dual interior point approach [5] and SQL (Sequential Quadratic Programming approach) [6] are proposed for the instant discovery of infeasibility. Robotics use an equilibrium condition [7], [8]. For a bio-inspired spider robot's leg posture and effective force distribution, the equilibrium condition is used in [7]. In [8], humanoid robots are subjected to the equilibrium condition in order to attain stable balance. Here we adapt to self-organized urban street networks the Metropolis algorithm. The coming to equilibrium distribution is carried out with MaxEnt by taking scale-freeness as prior hypothesis along with symmetry-conservation arguments [9]. A model for minimising the cost of collecting information while ensuring specified levels of demand are served at an acceptable level of reliability is proposed, and the characteristics of the proposed formulation are coupled with another planning objective and applied to identify optimal sites for information provision in a transportation system [10]. A method of equilibrium path analysis and stability analysis of an equilibrium state for a rigid origami is presented in [11], that have rigid flat faces connected by straight crease lines and can be folded and unfolded without deformation of its faces. An innovative approach is proposed to multi-objective optimization by integrating non-dominated sorting and critical equilibrium principles. [12]. Their method enhances the search for Pareto-optimal solutions by effectively navigating complex optimization landscapes. A Pareto-critical equilibrium is examined in detail as it relates to MOOPs with constraints [13]. Their research clarifies the complex relationship that exists between the feasibility of solutions in limited optimization settings and critical equilibrium conditions. Furthermore, optimization methods are enhanced by introducing an approach for using the Pareto-critical equilibrium condition to solve multi-objective optimization problems [14].

Multi-objective optimization problems (MOOPs) are global in real-world scenarios, where decisions involve optimizing multiple conflicting objectives simultaneously. Finding solutions that balance these objectives efficiently is crucial for various applications, including resource allocation, engineering design, and economic planning. However, traditional methods for solving MOOPs often face limitations when dealing with non-linearity and infeasible regions. Therefore, the proposed work deals with the infeasible regions where we need solutions for specific reasons, for instance, as discussed in the application section of this paper. Therefore, this research paper proposes a new Pareto-critical equilibrium condition (PCEC) specifically designed to address such challenges.

It may seem odd that optimal solutions can exist in the infeasible region. It's crucial to understand that in certain situations, these kinds of answers may still be applicable in the real world. It has always been a challenging problem to locate base stations in difficult-to-reach areas, frequently requiring a trade-off between achieving the best coverage and honoring practical constraints. This paradigm is challenged by the new equilibrium condition in such scenarios, an application known as the PCEC. It directs us to optimal solution that are in infeasible region. Moreover, another application of the proposed PCEC for the agents in multi-agent systems can also learn about the best way to distribute resources or tasks by finding the optimal solution, even if it is infeasible. Strategies that maximize results while adhering to environmental constraints can be established using this condition. Thus, the PCEC's potential to find solutions in the infeasible region while also recognizing its limitations offers a useful tool for resolving real-world MOOPs when practicality needs to compensate for limitations.

The research presented here offers a powerful and new approach for resolving non-linear MOOPs. Due to its ability to handle non-linearity, find solutions in infeasible regions, and provide global validity under convexity, the suggested PCEC offers a number of improvements over current techniques.

Several noteworthy advances in the field of multiobjective optimization have been contributed by this research: For multiobjective optimization problems (MOOPs), the Pareto-critical equilibrium condition (PCEC) provides a powerful and innovative method of finding the best possible solution. This novel idea removes uncertainty and simplifies the search process by establishing a necessary and sufficient condition for optimality, which is especially useful for situations involving convex objective functions. Furthermore, PCEC broadens the search space beyond the usually considered feasible region, making it possible to find optimal solutions that might lie outside of previously established bounds. This increases the likelihood of discovering novel and possibly better answers than those found with traditional methods. Additionally, PCEC ensures the worldwide validity of its solutions for convex problems, giving researchers a high level of confidence in their results. Additionally, by enabling the transformation of MOOPs into single-objective optimization problems, PCEC provides access to new research directions and wider application in a variety of fields, including the social sciences and engineering. In conclusion, PCEC offers improved search capabilities, global guarantees for convex problems, and fascinating opportunities for more study and application, making it a useful tool for researchers looking for optimal solutions in MOOPs.

This study describes the Pareto-criticality equilibrium condition, which states that nonlinear multiobjective optimisation problems are infeasible. The proposed Pareto-critical equilibrium condition becomes sufficient under the assumption of rigorous convexity. The recommended procedure is comprised of the steps described below; however, unlike the recommended procedure in step [1], the resultant points contain new PCEC.

(i) Define an unconstrained auxiliary MOOP.

(ii) Find the auxiliary problem's Pareto-critical point.

(iii) The resulting point is either a feasible point for the original problem or a point at which the infeasibility condition satisfies.

(iv) Points that satisfy the infeasibility condition also hold the PCEC. This indicates the creation of a new PCEC. When using a multi-agent system to search for a minimum, such Pareto-critical equilibrium conditions can be applied for a global minimum. For the points in this region, no prior PCEC was created.

The rest of the paper is carried out as follows: Section 2 presents notations and background materials. Section 3 presents the infeasibility condition (INF). The proposed Pareto critical equilibrium condition is explained in Section 4. Section 5 presents results and discussion with illustrated examples. Section 5 presents applications of the proposed condition, and Section 6 ends this paper with a conclusion.

## 2 Notations, backgrounds and statement of the problem

### 2.1 Notation

The following notations are used for vector arguments.

• P is used for Pareto-optimal set.
• Ω is used for set of feasible points.
• Λ is used for set of infeasible points.
• (≤) means that each of the first argument's coordinates is equal to or less than the second argument's corresponding coordinate. Similarly (<) is used for strictly less and (≥) is used for greater or equal to and (>) for strictly greater.
• (≺) each of the first argument's coordinates is equal to or less than the second argument's corresponding coordinate, and at least one of the first argument's coordinates is precisely smaller than the second argument's corresponding coordinate.
• $S(\overset{¯}{y})=\left[\nabla s_{1}(\overset{¯}{y})\;\nabla s_{2}(\overset{¯}{y})\;\nabla s_{3}(\overset{¯}{y})\;\nabla s_{4}(\overset{¯}{y})\ldots \nabla s_{p}(\overset{¯}{y})\right]$, the function's Jacobian matrix $s(\cdot ):R^{n}\mapsto R^{p}$ at $\overset{¯}{y}$.
• $T(\overset{¯}{y})=\left[\nabla t_{1}(\overset{¯}{y})\;\nabla t_{2}(\overset{¯}{y})\;\nabla t_{3}(\overset{¯}{y})\ldots \nabla t_{m}(\overset{¯}{y})\right]$, the function's Jacobian matrix $t(\cdot ):R^{n}\mapsto R^{m}$ at $\overset{¯}{y}$.

### 2.2 Multiobjective and constrained multiobjective optimization problems:

*Multiobjective Optimization Problems (MOOPs):*

MOOPs are optimization problems that involve optimizing multiple, often conflicting, objectives simultaneously. Unlike single-objective optimization, where there is one best solution, MOOPs have a set of Pareto-optimal solutions. These are solutions where no objective can be improved without worsening another.

$$\min_{y\in Y}\text{s}(y)=(s_{1}(y),s_{2}(y),...,s_{n}(y))\text{s.t.}y\in Y$$

where:

*y* is the vector of decision variables. s(y) is the vector-valued objective function with *n* objectives. *Y* is the feasible region.

*Constrained Multiobjective Optimization Problems (CMOPs):*

CMOPs are a type of MOOPs that also has constraints that limit the feasible solutions. These constraints can be applied to either the decision variables or the objective values.

$$\min_{y\in Y}\text{s}(y)=(s_{1}(y),s_{2}(y),...,s_{n}(y))\text{s.t.}g_{i}(y)\leq 0,\;i=1,...,m\;h_{j}(y)=0,\;j=1,...,p\;y\in Y$$

where:

$g_{i}(y)\leq 0$ are the inequality constraints. $h_{j}(y)=0$ are the equality constraints. Both types of constraints further restrict the feasible region *Y* of solutions.

### 2.3 Constrained optimization

In order to demonstrate the viability of the proposed Pareto-critical equilibrium condition, have a look at the optimization problem as stated by:

(1) $$\begin{array}{c} \min_{y}s(y)\\ \text{subject to: }t(y)\leq 0 \end{array}$$

in above, $s(\cdot ):R^{n}\mapsto R^{p}$ and $t(\cdot ):R^{n}\mapsto R^{m}$ are vector functions. The following is feasible solution set for this problem

(2) $$\Omega ≜\left\{y\in R^{n}\;|\;t(y)\leq 0\right\}$$

In particular, (1) is a single-objective optimization problem if p=1. However, if p>1, the problem (1) becomes a MOOP, and a feasible point $y\in R^{n}$ is said to be lead by another feasible point $\overset{¯}{y}\in R^{n}$ if $s(\overset{¯}{y})≺s(y)$. The solution set of (1)in the feasible region that are not dominated by any other point is referred to as the set P⊂Ω of feasible points. This set is called the efficient solution set, or the Pareto-optimal set. The general term for the solution set of a single-objective problem is P. From [1], we assume the following condition:

Assumption 2.1 From [1], assume $\left\{t_{1}(\cdot ),t_{2}(\cdot ),\ldots ,t_{k}(\cdot )\right\}$ is a subset of the constraint functions with k≤m, such that the set $\Omega_{c}\subset R^{n}$: defined by a non-empty compact set.

$$\Omega_{c}=\left\{y\;|\;t_{1}(y)\leq 0,\;t_{2}(y)\leq 0,t_{3}(y)\leq 0,\ldots ,\;t_{k}(y)\leq 0\right\}\;⋄$$

This kind of assumption is valid for a wide range of problems when a search needs to be done.

### 2.4 Pareto optimality

The primary objective in multiobjective optimization problems (MOOPs) is to generate solutions that balance competing objectives, referred to as Pareto-optimality, in order to achieve the best possible outcome.

Definition 2.1 Let $s(\cdot ):R^{n}\mapsto R^{p}$ be a function and $\Omega \subset R^{n}$ be a non-empty set of feasible solutions. A feasible solution $\overset{¯}{y}\in \Omega$ is said to be Pareto-optimal solution of Eq. (1) if and only if there does not exist any other feasible solution y∈Ω such that $s(y)≺s(\overset{¯}{y})$.

### 2.5 Kuhn-Tucker conditions for efficiency

Let $\lambda \in R^{p}$ and $\mu \in R^{m}$. The Kuhn-Tucker conditions of efficiency at a solution $\overset{¯}{y}$ of problem (1)as stated in [15], [16] is given by in Eq. (3):

(3) $$\text{(KTE)}\left\{ \begin{array}{c} S(\overset{¯}{y})\;\lambda +T(\overset{¯}{y})\;\mu \;=\;0\\ \lambda ≻0\;,\;\mu \geq 0\\ t(\overset{¯}{y})\;\leq \;0\\ \mu_{i}\;t_{i}(\overset{¯}{y})\;=\;0\;;\;\forall \;i=1,\ldots ,m \end{array}\right.$$

One important point to consider is that the KTE conditions for optimality in a single-objective scenario represent a specific instance of the more general Kuhn-Tucker conditions for optimality in multiobjective optimization problems (MOOP).

## 3 Infeasibility conditions

For any point $\overset{¯}{y}\in R^{n}$, one of the four possibilities must occur for (1).

**(a)** $\overset{¯}{y}\in P$, means that the Kuhn-Tucker required condition for efficiency satisfied:
**(b)** $\overset{¯}{y}\in \Lambda$, with Λ defined as the set of points for which hold:
  (4) $$\text{(INF)}\left\{ \begin{array}{c} \exists \;i\;|\;t_{i}(\overset{¯}{y})>0\\ T(\overset{¯}{y})\mu \;=\;0\\ \mu ≻0\\ t_{j}(\overset{¯}{y})<0\;\Rightarrow \;\mu_{j}\;=\;0 \end{array}\right.$$
  for some vectors of multipliers $\mu \in R^{m}$.
**(c)** $\overset{¯}{y}\in \Omega$ and $\overset{¯}{y}\notin P$.
**(d)** $\overset{¯}{y}\notin \Omega$ and $\overset{¯}{y}\notin \Lambda$.

## 4 Pareto-Critical Equilibrium Condition (PCEC)

If the two following conditions are met, a point is said to have the Pareto-critical equilibrium condition(PCEC).

1. The summation of all gradient vectors at a point, each with its own weight, results in zero.
  (5) $$\sum \mu_{i}▿\overset{ˆ}{t_{i}}=0\;\forall \;i=1,\ldots ,m$$
2. At some point, the summation of all torque vectors has a value of zero.
  (6) $$\sum \tau_{i}=0\;\forall \;i=1,\ldots ,m$$
  Varignon's theorem asserts that the total torque given in Eq. (6) resulting from multiple forces acting on a single point is equal to the torque generated by the sum (resultant) of these forces [17]. It is worth noting that this theorem is different from the geometrical theorem of the same name. Mathematically, we can express this relationship from Eq. (5) as follows:
  (7) $$\mu_{1}\times ▿{\overset{ˆ}{t}}_{1}+\mu_{2}\times ▿{\overset{ˆ}{t}}_{2}+\ldots \mu_{m}\times ▿{\overset{ˆ}{t}}_{m}=\mu_{i}\times (▿{\overset{ˆ}{t}}_{1}+▿{\overset{ˆ}{t}}_{2}+\ldots ▿{\overset{ˆ}{t}}_{m})=0$$
  In Eq. (7) $\mu_{i}$ are weight vector and $▿{\overset{ˆ}{t}}_{i}$are gradient vector.

The point is in Pareto-critical equilibrium when both conditions are met at $\overset{¯}{y}\in R^{n}$. E stands for the set of all such points that satisfy Pareto-critical equilibrium condition is given in Eq. (8).

(8) $$E=\left\{\overset{¯}{y}\in R^{n}:\;\;\overset{¯}{y}\;\;is\;\;an\;\;infeasible\;\;point\;\;for\;\;which\;\;PCEC\;\;hold\right\}$$

Points that meet the (KTE) criteria are Pareto-critical for problem (1). The condition (INF) is quite comparable to, as demonstrated by an auxiliary problem (KTE). See [1]. The following Eq. (9) describes the vector function defined in [1].

(9) $$\begin{array}{c} {\overset{ˆ}{t}}_{i}(y)=\left\{ \begin{array}{c} 0\;\;,\;\;\;\;\;\;\;\forall \;\;y\;|\;t_{i}(y)\leq 0\\ t_{i}(y)\;\;,\;\;\forall \;\;y\;|\;t_{i}(y)>0 \end{array}\right.\\ i=1,\ldots ,m \end{array}$$

The aforementioned definition leads to the auxiliary problem described below:

(10) $$\begin{array}{c} \min_{y}\overset{ˆ}{t}(y) \end{array}$$

The efficient solution set of (10) is denoted by the symbol B given in Eq. (11).

(11) $$B=\left\{y\in R^{n}\;|\;∄\;\overset{¯}{y}\in R^{n}\text{ such that }\overset{ˆ}{t}(\overset{¯}{y})≺\overset{ˆ}{t}(y)\right\}$$

Based on the Assumption 2.1, it's significant to remember that the following is conceivable:: B≠∅ and $B\subset \Omega_{c}$; [1].

The main version of this problem (without convexity) and the stronger version (convexity), respectively, are described in the following theorems extracted from [1].

Lemma 4.1 *[1]* *Consider an optimization problem defined by* (1) *and then:*

(12) $$\begin{array}{c} B=\Omega \neq \emptyset \Leftrightarrow (\Lambda \cap B)=\emptyset \\ (B\cap \Lambda )\neq \emptyset \Leftrightarrow \Omega =\emptyset \end{array}\;⋄$$

Lemma 4.2 *[1]* *Consider the problem of optimization defined by* (1) *, and assume that the functions* $s(\cdot ):R^{n}\mapsto R^{p}$ *and* $t(\cdot ):R^{n}\mapsto R^{m}$ *are convex. Then:*

(13) $$\begin{array}{c} B=\Omega \neq \emptyset \Leftrightarrow \Lambda =\emptyset \\ B=\Lambda \neq \emptyset \Leftrightarrow \Omega =\emptyset \end{array}\;⋄$$

As a result of Lemma 4.2, which holds true for convex instances and performed a search for Pareto-critical points $y_{a}\in B$, the search for a point inside the feasible set Ω of problem (1) may be described as multi-objective optimization on the auxiliary problem (10). If a point $y_{a}\in B$ has been discovered, then there are two potential outcomes: (i) $y_{a}\in \Omega$

(ii) If $y_{a}$ belongs to the set Λ, then it is also a part of E, which is the collection of points that satisfy the Pareto-critical equilibrium condition.

To discover a point $y_{a}$ within the set B is carried out in order to verify the proposed Pareto-critical equilibrium conditions. This point can result in an infeasibility certificate or a feasible point [1]. The generated points hold PCEC in the case of infeasibility.

It is sufficient to find a single Pareto-optimal solution to the auxiliary problem to establish the viability of the proposed PCEC. Below is a description of a scalarized variant of the multi-objective auxiliary problem (10).

(14) $$\begin{array}{c} \min_{y}\max_{i}\mu_{i}\;{\overset{ˆ}{t}}_{i}(y) \end{array}$$

The Pareto-optimal solution of (10) is each of the optimal solutions of (14). There is a weight vector $\mu_{i}$ that corresponds to each Pareto-optimal point $\overset{¯}{y}$ such that $\overset{¯}{y}$ is the optimum solution to (14); see [15].

Solutions to problem (14) either make the problem feasible or result in a certificate of infeasibility for problem (1). See [1]. When (1) is strictly infeasible, the consequence is:

Lemma 4.3 *[1]**Consider a solution*$y_{a}$*of problem*(14)*. Then:*

(15) $$y_{a}\notin \Omega \Rightarrow \Omega =\emptyset \;⋄$$

The proof in [1] confirms the infeasibility of the problem (1). If problem (1) is not feasible, the resulting points also satisfy the PCEC, which has applications to problems in the real world.

## 5 Results and discussions with illustrative examples

An easy computational framework has been established to verify the proposed PCEC. The non-linear Nelder Mead Simplex Method (NMSM) can be used to solve problem (14) because it is a single objective problem with non-differentiable functions. $y_{0}$ represents a random starting position. As a result, the non-linear problem has the value $\overset{¯}{y}$ as its solution. The Pareto-critical equilibrium condition will hold if the calculated solution, $\overset{¯}{y}$, is infeasible.

This section includes several sample test problems to illustrate the suggested algorithm. Each problem leads to the rigorous infeasibility of the original problem (1) being feasible. In the event of infeasibility, the PCEC will hold, and the discovered solution, $\overset{¯}{y}$, will belong to the set Λ=E. In Matlab [18], computational tests are put into practice. On a Pentium 2 Quad (Q6600) with RAM of 8GB and Windows 7 as its operating system, computational tests for each of the tested tasks were carried out. The algorithm is examined using the four Pareto-critical equilibrium conditions cases listed below. Random selection is used to choose the starting points.

Example 5.1 The optimization problem in this example involves three constraints and three agents, denoted as $\mu_{1}$, $\mu_{2}$, and $\mu_{3}$. These agents continuously share information via gradient vectors (communication) within restricted regions. If the problem is strict infeasible, the obtained solution points satisfy the PCEC.

$$\min_{y}s(y)\text{subject to:}\;t_{1}(y)={y_{1}}^{2}+3{y_{2}}^{2}-4\leq 0\;t_{2}(y)=-y_{1}+{y_{2}}^{2}\leq 0\;t_{3}(y)={y_{1}}^{2}+{\left(y_{2}-5\right)}^{2}-4\leq 0$$

The function s(y) in this illustration could have a single or multiple objectives.

For this, the vector function can be defined as follows;

$$\begin{array}{c} {\overset{ˆ}{t}}_{i}(y)=\left\{ \begin{array}{c} 0\;\;,\;\;\forall \;\;y\;|\;t_{i}(y)\leq 0\\ t_{i}(y)\;\;,\;\;\forall \;\;y\;|\;t_{i}(y)>0 \end{array}\right.\\ i=1,\ldots ,3 \end{array}$$

This vector function yields the following definition of the auxiliary problem:

$$\begin{array}{c} \min_{y}\overset{ˆ}{t}(y) \end{array}$$

That is,

$$\min_{y}\overset{ˆ}{t}(y)=\min\left(\begin{array}{c} {\overset{ˆ}{t}}_{1}(y)\\ {\overset{ˆ}{t}}_{2}(y)\\ {\overset{ˆ}{t}}_{3}(y) \end{array}\right)$$

Using minmax formulation we have

$$\min_{y}\max_{i}u_{i}{\overset{ˆ}{t}}_{i}(y)$$

**Figure 1:**
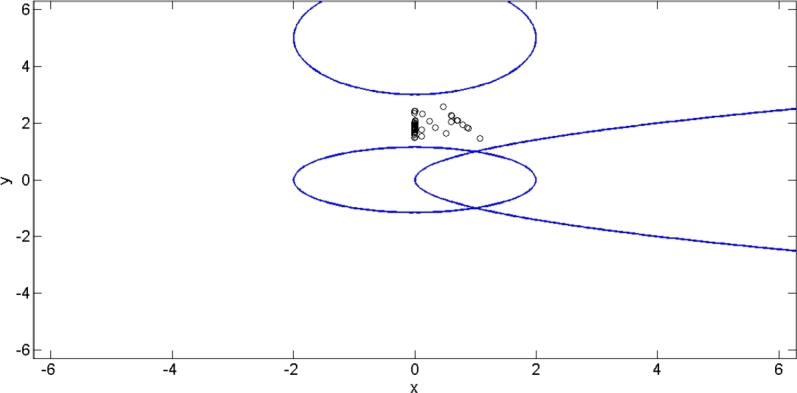
Solution points satisfying the PCEC.

The minmax problem has been solved by using Nelder Mead Simplex method [19]. The Fig. 1 illustrates the infeasibility of the problem and the PCEC satisfied by the obtained solutions. As a result, these solution points stand in for the shared objectives of agents $\mu_{1}$, $\mu_{2}$, and $\mu_{3}$ in the restricted area.

This example shows that if Ω=∅ and Λ≡E, then all solution points fall within the set Λ=E, which satisfies the PCEC.

Example 5.2 Let's consider the aforementioned example, but with only the first two constraints and two agents, denoted as $\mu_{1}$ and $\mu_{2}$.

$$\min_{y}s(y))\text{Subject to:}\;t_{1}(y)={y_{1}}^{2}+3{y_{2}}^{2}-4\leq 0\;t_{2}(y)=-y_{1}+{y_{2}}^{2}\leq 0$$

By following the same procedure, we can determine that the problem is feasible and has only one optimal solution.

**Figure 2:**
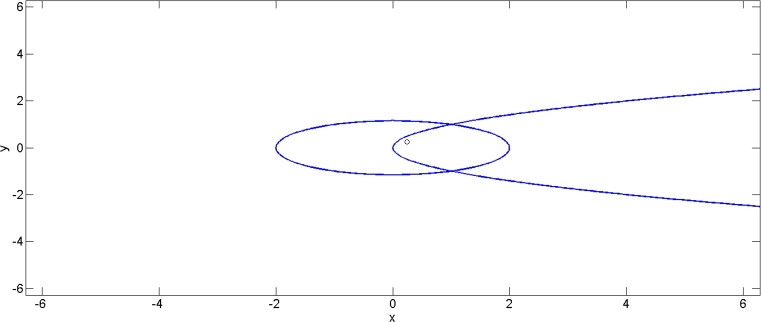
Shows that the problem has only one feasible solution.

Fig. 2 indicates that the problem is solvable and has a unique feasible solution. Specifically, we have A=Ω≠∅ and Λ=E=∅.

Example 5.3 Let's examine the aforementioned example, but with only the last two constraints.

$$\min_{y}s(y)\text{subject to:}\;g_{2}(y)={y_{1}}^{2}+3{y_{2}}^{2}-4\leq 0\;g_{3}(y)={y_{1}}^{2}+{\left(y_{2}-5\right)}^{2}-4\leq 0$$

**Figure 3:**
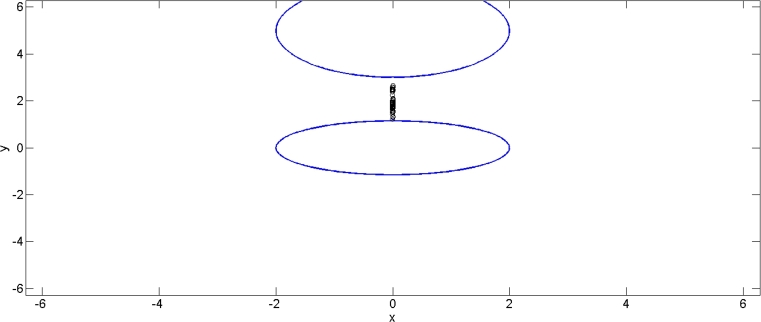
The dense region in the figure represents a set of infeasible solutions that satisfy the Pareto-critical equilibrium condition.

According to Fig. 3, the problem is strictly infeasible but meets the Pareto-critical equilibrium condition, as evidenced by the set of Pareto-critical solutions.

Example 5.4 The problem with 4 constraints and 4 agents $\mu_{1}$, $\mu_{2}$, $\mu_{3}$ and $\mu_{4}$ respectively, as presented in [6], can be expressed as an optimization problem.

$$\min_{y}s(y)\text{subject to:}\;t_{1}(y)=y_{1}+{y_{2}}^{2}+1\leq 0\;t_{2}(y)=-{y_{1}}^{2}+{y_{2}}^{2}+1\leq 0\;t_{3}(y)={y_{1}}^{2}+y_{2}+1\leq 0\;t_{4}(y)={y_{1}}^{2}+y_{2}+1\leq 0$$

**Figure 4:**
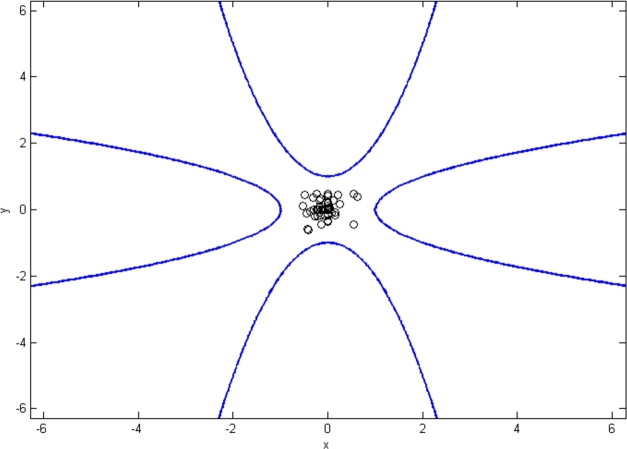
The Pareto-critical equilibrium conditions are satisfied by all infeasible solutions in this case.

A beam of infeasible solution points is illustrated in Fig. 4, indicating strict infeasibility of the problem. Nonetheless, all solution points are found to satisfy the PCEC for agents $\mu_{1}$, $\mu_{2}$, $\mu_{3}$ and $\mu_{4}$.

Example 1 The following is an optimization problem with six constraints and six agents:

$$\min_{y}s(y)\text{Subject to:}\;t_{1}(y)=-y_{1}+y_{2}^{2}\leq 0\;t_{2}(y)=y_{1}^{2}-y_{2}^{4}\leq 0\;t_{3}(y)=y_{1}^{2}+3y_{2}^{2}-4\leq 0\;t_{4}(y)=y_{1}^{2}-y_{2}+1\leq 0\;t_{5}(y)=y_{1}^{2}+{\left(y_{2}-5\right)}^{2}-4\leq 0\;t_{6}(y)=2y_{1}^{2}y_{2}^{\exp y_{1}}-y_{2}^{4}\leq 0$$

Example 2 Problem with seven agents and seven constraints is given by:

$$\min_{y}s(y)\text{Subject to:}\;t_{1}(y)=y_{1}^{2}-y_{2}^{4}\leq 0\;t_{2}(y)=-y_{1}+y_{2}^{2}\leq 0\;t_{3}(y)=y_{4}^{y_{3}}e^{y_{4}}\leq 0\;t_{4}(y)=2y_{1}^{2}y_{2}^{\exp y_{4}}-y_{2}^{4}\leq 0\;t_{5}(y)=+y_{1}^{2}+y_{2}+y_{3}^{5}+y_{4}^{2}+1\leq 0\;t_{6}(y)=y_{1}^{2}+{\left(y_{3}-5\right)}^{2}-4\leq 0\;t_{7}(y)=y_{1}^{2}+3y_{2}^{2}-4\leq 0$$

Three further examples, known in Byrd et al. [6], as Example 1, Example 3, and Example 5, are also included in this paper and are referred to as Byrd 1, Byrd 2, and Byrd 3, respectively.

Table 1, Table 2 present the computation outcomes for batches of 30 runs in four different scenarios, where the maximum number of iterations (iterMax) is set to 30, 50, 200, and 500.

**Table 1:** Computational findings for the Byrd 1, Byrd 2 and Byrd 3 problems.

	Byrd 1		Byrd 2		Byrd 3	
iter	30	50	30	50	30	50

FC	0	0	0	0	0	0
PEC = IC	30	30	28	30	30	30
NC	0	0	2	0	0	0
$\#\overset{¯}{y}$	1,00	1,00	1,86	2,03	1,00	1,00

**Table 2:** Computational findings for Example 1 and Example 2.

		Example 1			Example 2		Example 1
iter	30	50	200	30	50	200	500

FC	0	0	0	0	0	0	0
PEC = IC	0	15	26	6	29	30	30
NC	30	15	4	24	1	0	0
$\#\overset{¯}{y}$	3,00	36,93	88,46	2,90	13,83	15,36	74,73

In computational findings tables, lines “FC” and “IC = PEC” respectively denote the number of feasibility certificates and the number of infeasibility certificates. Where “PEC” stands for the sites where Pareto-critical equilibrium conditions are met. Line “NC” shows how many iterations were required to earn any certificate. The average quantity of solutions for each instance is shown in line “$\#\overset{¯}{y}$”.

The algorithm for six and seven constraints is explained in this paper using Example 1 and Example 2 explained above. In other words, the procedure works for any quantity of constraints. The goal of the proposed strategy is to determine whether any solution $\overset{¯}{y}$ is feasible, infeasible, or inconclusive. The developed solutions satisfy the Pareto-critical equilibrium condition in the case of an infeasibility certificate.

Analyzing the results in Tables 1 and 2, specifically at the point of maximum iterations, the experimental results demonstrate that the proposed algorithm has the capability to provide certificates of feasibility or infeasibility for the given optimization problems. The solution points also meet the Pareto-critical equilibrium condition in the case of infeasibility. For instances Byrd 1 and Byrd 3, the algorithm gives 100% accuracy when iterMax=30. With 50 iterations, the algorithm finds infeasibility in 50% of Example 1 executions and in 99% of Example 2 executions. When a maximum of 500 iterations are used, the method provided certificates for all tested cases.

## 6 Applications of Pareto-critical equilibrium conditions

**Application: 01**

**Establishing a Base Station in Proper Location using Pareto-Critical Equilibrium Conditions:**

**Figure 5:**
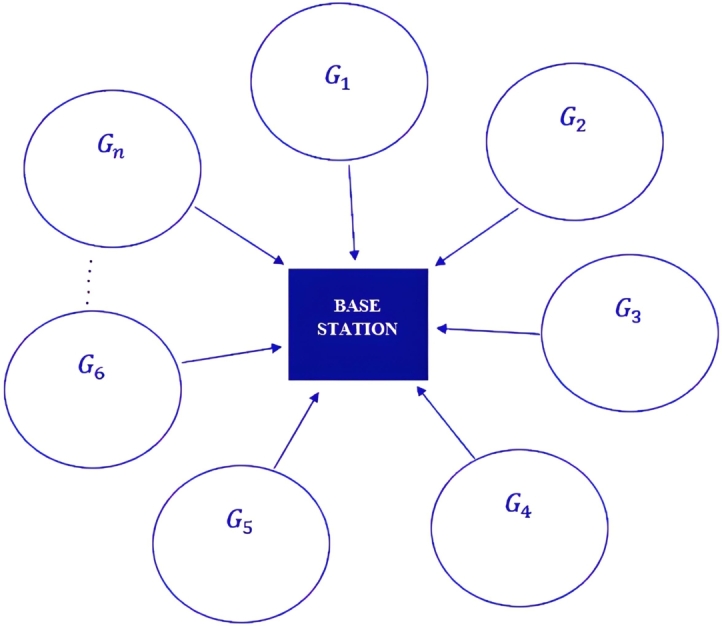
A beamforming system for downlink transmission with several multicasts. The base station has *K* antennas, *n* groups, and a total of *G* = *G*_1_,*G*_2_,....*G*_*n*_ single-antenna receivers.

Consider a downlink transmit beamforming system, represented in Fig. 5, where a base station equipped with *K* transmit antennas intends to transmit *n* different data streams to *G* single antenna towns, over a shared frequency range. The towns are divided into *n* groups $G_{i},i=1,2,...,n$, with each group having clients interested in the same data stream. The broadcasting data stream and beamforming vector for the $i^{th}$ group are represented by $\tau_{i}$ and $\mu_{i}$, respectively. The broadcast signal at the base station is the sum of the product of the beamforming vectors and data streams, given by $\sum_{i=1}^{n}\mu_{i}\tau_{i}$. Here, it is important to note that each group is assigned a unique beamforming vector to ensure that the clients in the same group receive the desired data stream.

1. All gradient vectors at point *y* have a weighted total that is equal to zero:
  $$\sum \mu_{i}▿\overset{ˆ}{t_{i}}=0\;,\forall \;i=1,\ldots ,n,$$
2. At point *y*, the summation of all torque vectors has a value of zero.
  $$\sum \tau_{i}=0\;,\forall \;i=1,\ldots ,n$$

Example 6.1 Consider the following optimization problem with four towns (constraints). Gradient vectors along the constrained zones are used by these towns to receive beamforming (signals). The discovered solution sites satisfy the Pareto-critical equilibrium condition in the strict case of problem infeasibility.

$$\min_{y}s(y)\text{subject to:}\;t_{1}(x)=2{y_{1}}^{2}+{\left({y_{2}}^{2}+2\right)}^{2}-8\leq 0\;t_{2}(x)=2{y_{1}}^{2}+{\left({y_{2}}^{2}-5\right)}^{2}-4\leq 0\;t_{3}(x)={\left(3y_{1}+9\right)}^{2}+{\left(2y_{2}-6\right)}^{2}-20\leq 0\;t_{4}(x)={\left(4y_{1}-10\right)}^{2}+{\left(3y_{2}-7\right)}^{2}-40\leq 0$$

The function s(y) in this illustration could have a single or multiple objectives.

For this, the vector function can be defined as follows;

$$\begin{array}{c} {\overset{ˆ}{t}}_{i}(y)=\left\{ \begin{array}{c} 0\;\;,\;\;\forall \;\;y\;|\;t_{i}(y)\leq 0\\ t_{i}(y)\;\;,\;\;\forall \;\;y\;|\;t_{i}(y)>0 \end{array}\right.\\ i=1,\ldots ,4 \end{array}$$

This vector function yields the following definition of the auxiliary problem:

$$\begin{array}{c} \min_{y}\overset{ˆ}{t}(y) \end{array}$$

That is,

$$\min_{y}\overset{ˆ}{t}(y)=\min\left(\begin{array}{c} {\overset{ˆ}{t}}_{1}(y)\\ {\overset{ˆ}{t}}_{2}(y)\\ {\overset{ˆ}{t}}_{3}(y)\\ {\overset{ˆ}{t}}_{4}(y) \end{array}\right)$$

Using minmax formulation we have

$$\min_{y}\max_{i}u_{i}{\overset{ˆ}{t}}_{i}(y)$$

The minmax problem has been solved by using Nelder Mead Simplex method [19]. The figure illustrates the infeasibility of the problem and the PCEC satisfied by the obtained solutions. Since all four towns in the confined area share these solution points, they serve as their overall objectives.

This example shows that if Ω=∅ and Λ≡E, then all solution points fall within the set Λ=E, which satisfies the PCEC given in Eqs. (5) and (6). That is:

1. All gradient vectors at some point have a weighted total that is equal to zero:
  $$\sum \mu_{i}▿\overset{ˆ}{t_{i}}=0\;\forall \;i=1,\ldots ,m$$
2. At some point, the summation of all torque vectors has a value of zero.
  $$\sum \tau_{i}=0\;\forall \;i=1,\ldots ,m$$

**Figure 6:**
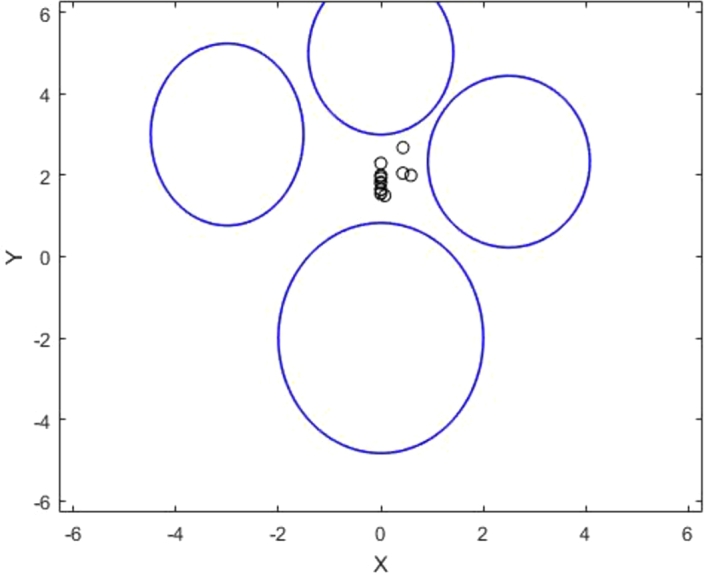
Solution points satisfying the PCEC.

The solved example considers 4 towns as constraints, each having a convex shape. To ensure the minimum distance between the base station and all the towns, we need to place the base station at an appropriate location. As shown in Fig. 6, none of the solution points are feasible, thus necessitating the placement of the base station at the minimum distance to all 4 towns.

Example 6.2 Consider the following optimization problem with 6 towns (constraints). Gradient vectors along the constrained zones are used by these towns to receive beamforming (signals). The discovered solution sites satisfy the Pareto-critical equilibrium condition in the strict case of problem infeasibility.

$$\min_{y}s(y)\text{subject to:}\;t_{1}(y)=2{y_{1}}^{2}+{\left({y_{2}}^{2}+2\right)}^{2}-4\leq 0\;t_{2}(y)=2{y_{1}}^{2}+{\left({y_{2}}^{2}-5\right)}^{2}-4\leq 0\;t_{3}(y)={\left(4y_{1}-10\right)}^{2}+{\left(y_{2}-10\right)}^{2}-30\leq 0\;t_{4}(y)={\left(4y_{1}+10\right)}^{2}+{\left(y_{2}-10\right)}^{2}-30\leq 0\;t_{5}(y)={\left(4y_{1}+10\right)}^{2}+{\left(3y_{2}+1\right)}^{2}-30\leq 0\;t_{6}(y)={\left(4y_{1}-10\right)}^{2}+{\left(3y_{2}+1\right)}^{2}-30\leq 0$$

Using the same method as before, we can apply the procedure to six towns acting as constraints.

**Figure 7:**
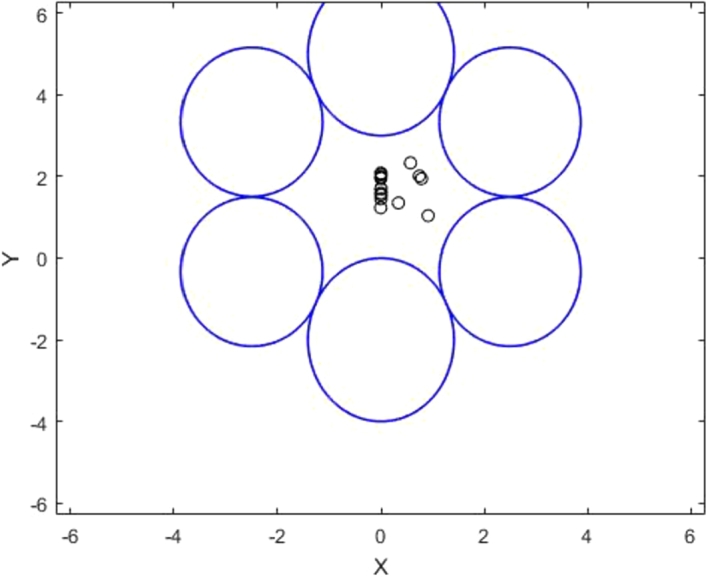
The solution points that holds the PCEC.

The Pareto-critical solutions in Fig. 7 demonstrate that the problem is strictly infeasible and satisfy the PCEC.

1. The summation of all gradient vectors at a point, each with its own weight, results in zero.
  $$\sum \mu_{i}▿\overset{ˆ}{t_{i}}=0\;\forall \;i=1,\ldots ,6$$
2. At a particular point, the summation of all torque vectors has a value of zero.
  $$\sum \tau_{i}=0\;\forall \;i=1,\ldots ,6$$

In the solved example, six towns are assumed. As every town has a convex shape, we would locate a base station there so that it is the shortest distance possible from every town. We may construct a base station at the closest possible location to each of the four towns because it is obvious from Fig. 7 that none of the solution points are feasible.

**Application: 02**

**Global Information Collection in a Multi-agent System using the Pareto-critical Equilibrium Condition**

The fundamental components of a multi-agent system are individual agents who work in cooperation, exchanging knowledge and information, to accomplish either their own objectives or a global shared goal. The scientific community provides an illustration of how a pluralistic community might operate [20]. If answers to problems are developed locally, they are shared with other issue solvers so they can be questioned, assessed, and improved [21]. Several real-world issues have to do with task allocation by giving an agent tasks and resources for solving issues. It improves the problem-solving abilities of problem-solving agents, which is the first of its two benefits. Also, it increases the likelihood that a problem will be solved consistently by reducing potential disagreements that could lead to time-consuming processes [22].

From an application standpoint, a workable solution of (1) will show the area that is serviced by certain agents. Gradient vectors serve as a means of communication among agents in a multi-agent system, allowing them to work together towards shared objectives. However, if the problem leads to infeasibility as per (1), the solution points must satisfy the Pareto critical equilibrium condition at certain points.

That is task which is equal to agents plus communications (Task = Agents + Communications).

To find their common objective, all $\mu_{i}$ agents communicate via gradient vectors $▿\overset{ˆ}{t}$. A certain moment will see the resultant goal hold Preto-critical equilibrium.

## 7 Conclusion

This paper introduces a new Pareto-critical equilibrium conditions (PCEC) for non-linear optimization problems in an auxiliary multi-objective optimization problem (MOOP). When the original problem is strictly infeasible, this new condition is utilized as the minimum point for multi-agent systems. The proposed Pareto-critical equilibrium condition is necessary (and sufficient under certain convexity assumptions) for optimization problems of finite dimensions. By defining an appropriate auxiliary vector function, the search for the feasible set can be expressed as the search for a point that satisfies the Pareto-critical condition for this auxiliary problem. Once a Pareto-critical point is discovered with respect to the auxiliary problem, it either offers a feasible solution for the original problem or establishes an infeasibility certificate that is globally valid for convex problems. The resulting infeasibility certificate satisfies the Pareto-critical equilibrium condition and has practical applications in real-world optimization problems, including the identification of an optimal location for a base station and problems involving multi-agents with specific objectives. The foundation for applying PCEC to various multiobjective optimization problems in real life is laid by this research. In the future, PCEC's applicability in a variety of contexts, including multi-agent coordination and the discovery of impractical solutions in complex engineering systems, will be investigated, along with modifications to handle non-convex problems.

## Funding

This research was supported by the Department of Mathematics, 10.13039/501100004684Abdul Wali Khan University Mardan 23200, Pakistan, and the 10.13039/100007630College of Engineering and Informatics, National University of Ireland, Galway, 91 CF50, Ireland.

## CRediT authorship contribution statement

**Shakoor Muhammad:** Formal analysis, Conceptualization. **Abdul Rehman:** Writing – review & editing, Methodology, Investigation. **Amjad Iqbal:** Software, Formal analysis. **Taimur Ali:** Validation, Writing – review & editing. **Faisal Khan:** Funding acquisition, Validation.

## Declaration of Competing Interest

The authors declare that they have no known competing financial interests or personal relationships that could have appeared to influence the work reported in this paper.

## Data Availability

No data available for this manuscript.
